# Christmas at the Hospitals

**Published:** 1888-01-28

**Authors:** 


### Christmas at the Hospitals.

(By our Commissioner and Correspondents.)

### ST. MARY'S, PADDINGTON.

A very pleasant concert, intended for the amusement of patients and nurses, took place in the board-room of this hospital on the evening of Thursday, the 11th inst. The majority of the performers were officials of the institution, which seems to be unusually rich in musical talent. A number of nurses gave some very tuneful part-singing, and the matron, Miss Medill, took part with Miss Lockett in Balfe's sprightly duet, "I Know a Maiden Fair to See." Among the gentlemen connected with the hospital, special mention must be made of the secretary, Mr. Thomas Ryan, whose rendering of "The Wolf" showed a practised and flexible execution not often attained by a bass voice; and Dr. Maguire, who played a "Bourrée" of Bach's with grace and vigour. Of the visitors who assisted in the performance, special plaudits were received by Miss Campbell, who sang two songs with excellent voice and a clear enunciation of the words, as pleasing as it is rare; and Mr. Sydney Teversham, whose recitations delighted the audience greatly. His rendering of "Jim Blain's Ram Story," a delightful piece of inconsequence, convulsed his hearers with laughter; as did Mr. Sydney Causton's musical sketch, which depicted very cleverly the humours of a country penny reading. That any penny reading was quite so funny we may doubt; but Mr. Causton had condensed the characteristics of many into this typical one, which well deserved the cordial reception it received. After the concert the performers and some of the guests assembled in Miss Medill's room, where all expressed the pleasure their share in the entertainment had afforded them.

### CONGLETON COTTAGE HOSPITAL.

Through the generosity of a large number of friends, Miss Wagnell, the kind-hearted matron of the above institution, was enabled on Wednesday last to give a grand tea and treat to the inmates and about two hundred poor children in the Congleton Cottage Hospital. There was provided an abundance of ham and tongue sandwiches, currant and spice bread, buns, preserves, etc., the tables being presided over by Mrs. and Miss Besant, Mrs. and Miss Condor, Miss Washington, Mrs. and Miss Hall, and Miss Bartlett (Chapel Street), Mrs. Hall (Market Place), and the Misses Reade; Mr. A. C. Condor, J.P., Mr. J. Hall (Chief Constable), and Mr. A. Hall rendering effective services as waiters. After ample justice had been done to the good things provided, the children adjourned to the day-room, where Mr. Condor had a magic lantern, which he manipulated with the assistance of Mr. A. Hall, to the great delight of the youngsters, who expressed their feelings by frequent roars of laughter. The Rev. J. M. Bannerman gave a short address, and the choir of St. Stephen's Church sang a selection of carols beautifully, after which the inmates and children were presented—every one—with warm articles of clothing, together with toys, a bag of sweets, oranges, apples, buns, nuts, etc., all going away highly delighted with and full of gratitude to the matron for the entertainment she had so kindly provided for them.

### LADIES' CHARITY SCHOOL, POWIS GARDENS, NOTTING HILL.

Mr. H. C. Burdett presented the prizes at this institution on Thursday, the 19th inst. In the course of a short address Mr. Burdett gave a sketch of the history of this charity, which was established in 1702 for the education, maintenance, clothing, and training in domestic service of the daughters of respectable parents in necessitous circumstances Dr. Johnson was one of the first to recognise the good work done by this school, and he wrote to Mrs. Thrale, who supported it, "Whatever reasons you have for frugality it is not worth while to save a guinea a year by saving it from public charity." The present patron of the institution is her Majesty the Queen, who never allows her name to be connected with any institution of whose usefulness she is not convinced. Mr. Burdett pointed out that while other labour markets were overstocked with workers, in that of domestic service the demand for competent help always exceeded the supply. The field of domestic service probably offered more prizes at the present day than any other, provided that those who chose it acted up to the standard laid down by Carlyle, which included faithful obedience, modesty, humility, and correct moral conduct. To this list Mr. Burdett added thoroughness and conscientiousness in the discharge of their duties. The speaker also explained how he had come to know of the existence of the school by sitting near the children in church, and on inquiring into its constitution felt surprised that he, who considered himself well acquainted with the charities of London, should have been so long ignorant of an institution which did a great deal of good work in a quiet way. He expressed the satisfaction he felt, being a medical man in all but the name, at seeing the admirable physical condition of the children, and dwelt on the necessity for attending carefully to all matters of hygiene. Finally he warned the girls that, though they could not hope in this world to escape being misunderstood and misrepresented, they would ultimately, if they did their duty, be rewarded by the respect and affection of those whom they served. After Mr. Burdett's remarks were concluded, and a vote of thanks to him moved by Miss A. C. Moore, the hon. secretary, the children were amused by witnessing a conjuring entertainment, and by a Christmas tree. The following is a list of the prizewinners, with the names of the ladies who gave the prizes:—

House-work.—1st. Ada Spivey, umbrella, given by Miss Whiting; 2nd. Louie Bonner, workbox, Miss Whiting; 3rd. Marion Flower, writing case, Mrs. Arthur Miles.

Needlework.—1st. Ellen Watts, book, given by Miss Hesba Stretton; 2nd. Elizabeth Forrester, book, Mrs. Moore; 3rd. Jane Stuart, book, Miss Hesba Stretton.

Arithmetic.—Standard IV. Kate Moss, book, given by Miss Moore; Standard V. Mabel Naish, book, [illegible]iss Moore.

Lessons and Behaviour.—1st. Jane Worthan, book, given by Miss H. Stretton; 2nd. Alice Rickards, book, Miss H. Stretton.

General Neatness.—Annie Dowden, book, given by Miss A. C. Moore.

General Good Conduct.—Mary Leatherdale, book, given by Mrs. A. Miles.

